# T-cell engagers in cancer immunotherapy: mechanisms, challenges, and future perspectives

**DOI:** 10.3389/fimmu.2026.1755512

**Published:** 2026-06-03

**Authors:** Kaaviyaa Muthughavi, Manoj Khokhar, Hem Chandra Jha, Rajan Kumar Pandey

**Affiliations:** 1 School of Biochemistry and Cell Biology, University College Cork, Cork, Ireland; 2 Department of Biochemistry, All India Institute of Medical Sciences, Jodhpur, Rajasthan, India; 3 Department of Biosciences and Biomedical Engineering, Indian Institute of Technology Indore, Madhya Pradesh, India; 4 Department of Medical Biochemistry and Microbiology (IMBIM), Uppsala University, Uppsala, Sweden

**Keywords:** bispecific antibodies, cancer immunotherapy, CAR-T-cell, T-cell engagers, tumor microenvironment

## Abstract

T-cell engagers (TCEs) are a rapidly evolving class of cancer immunotherapies that redirect cytotoxic T cells to tumor-associated antigens independently of MHC presentation. This review outlines the development, structural formats, and therapeutic potential of various TCE platforms, including bispecific (BiTEs) and trispecific T-cell engagers (TriTEs), dual affinity re-targeting (DART), and other emerging formats. We also highlight the clinical success in treating hematologic malignancies as demonstrated by agents such as blinatumomab and teclistamab. We also discussed the ongoing challenges in solid tumors, such as antigen heterogeneity and the immunosuppressive tumor microenvironment. A comparative analysis with CAR-T cell therapies is provided, with a focus on efficacy, safety, toxicity, resistance, and cost. Strategies to increase specificity and reduce toxicity, such as tumor-selective activation (e.g., XPATs), CD3 affinity tuning, and multifunctional constructs, are discussed. Future directions include AI-driven design, synthetic biology, and potential applications beyond oncology, including autoimmune and infectious diseases. As scalable, off-the-shelf therapeutics, T-cell engagers are poised to become essential tools in personalized and accessible cancer treatment.

## 1 Background

Cancer is a leading cause of premature death worldwide (1). Over the next 50 years, its global burden is expected to rise significantly due to demographic shifts, such as population aging and growth, which affect cancer incidence trends across regions. If current patterns persist, the overall cancer incidence could double by 2070 compared with 2020 (2). Conventional cancer treatments typically involve surgical tumor removal, followed by radiotherapy or chemotherapy. Among these methods, surgery is most effective in the early stages of disease progression. However, radiation therapy can harm healthy cells, organs, and tissues. While chemotherapy has helped reduce morbidity and mortality, it also affects healthy, rapidly dividing cells. A major challenge with chemotherapy is drug resistance, where initially suppressed cancer cells develop resistance owing to reduced drug uptake and increased drug efflux. Additionally, conventional chemotherapy has limitations such as dose selection difficulties, a lack of specificity, rapid drug metabolism, and harmful side effects (3). As a result, cancer immunotherapy has become increasingly popular among researchers in recent years.

Immunotherapy uses the body’s own immune system to combat cancer, which has led to groundbreaking treatment strategies and remarkable clinical advancements (4). In this treatment approach, T cells play a crucial role, with the ability to recognize and destroy cancer cells (5). T-cell-based cancer immunotherapies can be classified into two categories based on their mechanisms of action: those that counteract immunosuppressive factors, such as immune checkpoint inhibitors (ICIs), and those that amplify immunostimulatory pathways, including chimeric antigen receptor (CAR) T cells and T-cell-engaging bispecific antibodies (BsAbs) (6).

The development of BsAbs began in the 1960s, with significant advancements such as hybridoma technology, which enabled large-scale production of monoclonal antibodies. In 1983, the introduction of hybrid-hybridoma (quadroma) technology led to the creation of the first functional BsAbs. Later, in 1996, the development of knobs-into-holes (KiH) technology provided a reliable method for developing heterodimeric antibodies, paving the way for the development of modern T-cell engagers (TCEs). These therapies are designed to direct T cells to cancer cells more effectively, improving treatment outcomes for patients with hematological malignancies (7).

TCEs are synthetic immunoglobulin fragments or full-length molecules designed with dual affinity for CD3 and tumor-associated antigens (TAAs). They work by directly activating T cells at the site of tumor cells, leading to a rapid antitumor immune response (8). Beyond tumor cell-surface antigens, TCEs can also be designed to redirect T cells against stromal components of the tumor microenvironment, including cancer-associated fibroblasts (CAFs) via fibroblast activation protein (FAP) and tumor-associated macrophages (TAMs) via CD206 and folate receptor β, which are critical drivers of tumor progression, immune suppression, and therapeutic resistance (9, 10). These BsAbs are designed to bind both tumor-associated antigens on cancer cells and CD3 molecules on T cells. This dual binding mechanism activates T cells, increasing their ability to destroy tumor cells. Replacing the anti-CD3 domain with an anti-CD16 antibody allows NK cell recruitment. Additionally, the inclusion of a third binding domain can improve tumor targeting and enhance immune-cell activation, thereby strengthening the immune response against cancer (11). TCEs were developed to overcome the limitations of monoclonal antibody-based therapies, which do not engage T cells directly.

**Figure 1:**
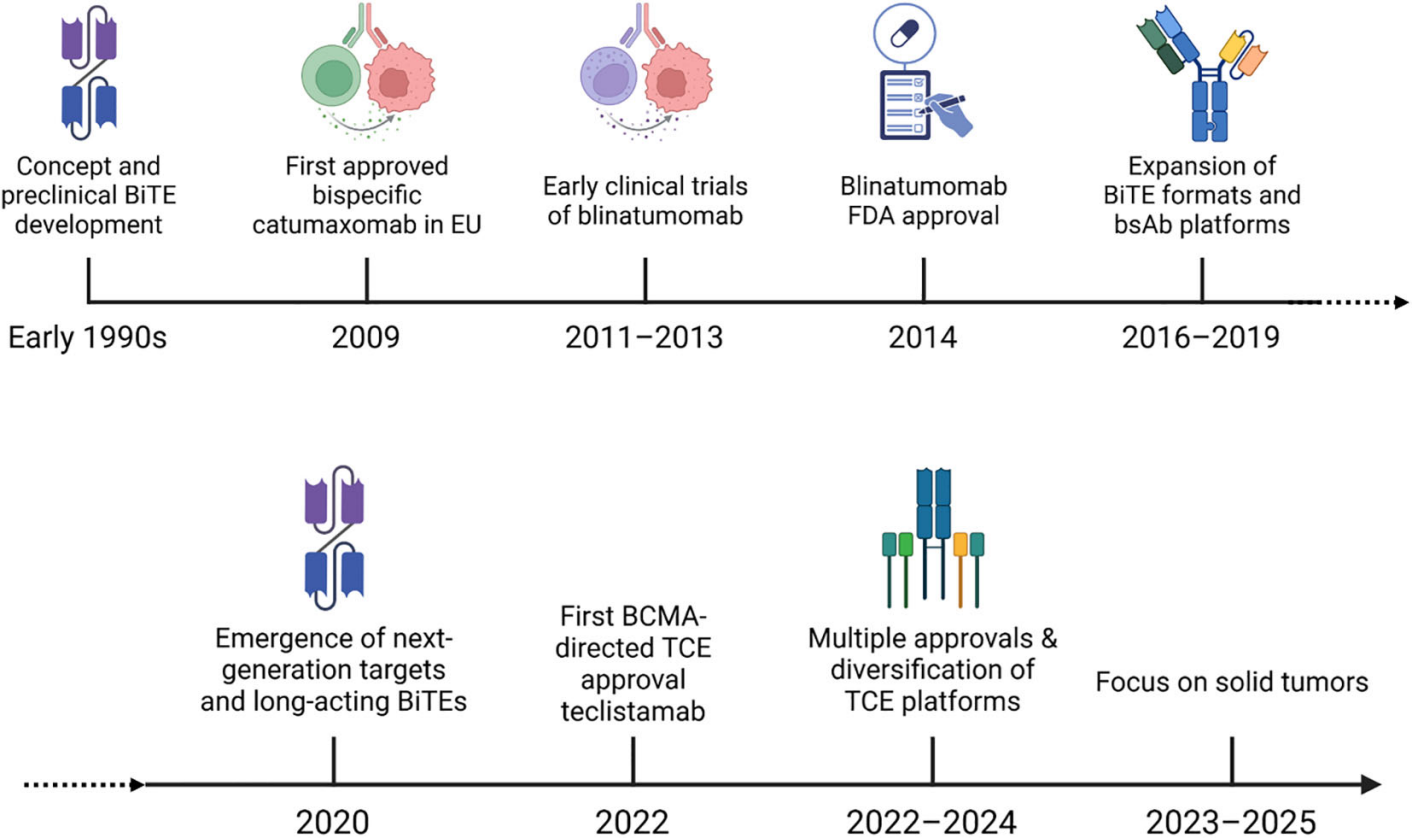
Chronological milestones of T-cell engagers in cancer immunotherapy. This figure presents a concise timeline summarizing the key developmental phases of T-cell engagers, including bispecific and trispecific antibody formats.

All BsAbs and trispecific antibodies (TsAbs) approved or in clinical development for hematologic malignancies are immune cell engagers (ICEs) (**Table 1**). These molecules typically have one specificity, i.e., one domain for recruiting T cells (TCEs) or NK cells (NKCE) and another for binding to a TAA. As of July 2023, nine BsAbs have been approved in the EU or the US, with two under regulatory review and four in late-stage development. Additionally, two studies were approved in China and Japan. Among these, nine are TCEs that target hematologic malignancies, including five approved (blinatumomab, mosunetuzumab, teclistamab, epcoritamab, and glofitamab), two under review (talquetamab, elranatamab), and two in late-stage trials (odronextamab, linvoseltamab) (11). The chronological milestones in the development of T-cell engagers for cancer immunotherapy are presented in **Figure 1**.

**Table 1:** Overview of approved and investigational CD3-redirecting T- cell engagers, including targets, molecular format, primary indications, regimens, and clinical development status (as of 2025).

Agent/trade name	Target (tumor x CD3)	Format/key feature	Primary indication	Regimen	Approval/clinical status (as of 2025)	Regulatory/trail source
Blinatumomab (Blincyto^®^)	CD19 × CD3	BiTE (scFv–linker–scFv)	B-cell precursor ALL (adult & pediatric)	Continuous intravenous infusion	FDA 2014, EMA 2015 approved	FDA, EMA
Teclistamab (Tecvayli^®^)	BCMA × CD3	IgG4 bispecific antibody​	Relapsed/refractory multiple myeloma	subcutaneous injection	FDA 2022, EMA 2022 approved	FDA, EMA
Talquetamab (Talvey^®^)	GPRC5D × CD3	IgG4 bispecific antibody	Relapsed/refractory multiple myeloma after ≥4 lines	subcutaneous injection	FDA 2023, EMA 2023 approved	FDA, EMA
Elranatamab (Elrexfio^®^)	BCMA × CD3	IgG2a bispecific antibody	Relapsed/refractory multiple myeloma	subcutaneous injection	FDA 2023, EMA 2023 approved	FDA, EMA
Epcoritamab (Epkinly/Tepkinly)	CD20 × CD3	Fc-silent bispecific antibody	Relapsed/refractory LBCL (≥ 2 prior lines); FL	subcutaneous injection	FDA 2023 (LBCL), FDA 2024 (FL); EMA 2023 (LBCL/FL) approved	FDA, EMA
Glofitamab (Columvi^®^)	CD20 × CD3	2:1 IgG-like bispecific antibody	Relapsed/refractory DLBCL and LBCL	intravenous infusion	FDA 2023, EMA 2023 approved	FDA, EMA
Mosunetuzumab (Lunsumio^®^)	CD20 × CD3	Full-length bispecific IgG antibody	​ Relapsed/refractory FL	intravenous infusion	FDA 2022, EMA 2022 approved	FDA, EMA
Tarlatamab (Imdelltra^®^)	DLL3 × CD3	Half-life-extended BiTE format	Extensive-stage small-cell lung cancer	intravenous infusion	FDA 2024, MHRA (UK) 2025 approved; no EMA approval	FDA, MHRA
Linvoseltamab (Lynozyfic™)	BCMA × CD3	Bispecific IgG4 antibody	Relapsed/refractory multiple myeloma	intravenous infusion	FDA 2025, EMA 2025 approved	FDA, EMA
Cevostamab	FcRH5 × CD3	Full-length bispecific IgG antibody	Relapsed/refractory multiple myeloma	intravenous infusion	Phase Ib ongoing (NCT04910568)	ClinicalTrials.gov
Etentamig ABBV-383 (TNB-383B)	BCMA × CD3	Low-CD3-affinity bispecific antibody	Relapsed/refractory multiple myeloma	intravenous infusion	Phase III ongoing (NCT05396885)	ClinicalTrials.gov
Plamotamab (XmAb13676)	CD20 × CD3	XmAb^®^ bispecific antibody	Non-Hodgkin lymphoma (DLBCL, FL, MCL)	Intravenous infusion and subcutaneous injection	Phase Ib ongoing (NCT02924402)	ClinicalTrials.gov
Cibisatamab	CEA × CD3	2:1 bispecific antibody	CEA-positive colorectal and solid tumors​	Intravenous infusion	Program discontinued (formerly Phase I/Ib)	ClinicalTrials.gov (NCT03866239)
ERY974	GPC3 × CD3	IgG-like bispecific antibody	Hepatocellular carcinoma (GPC3-positive)	Intravenous infusion	Phase I (NCT05022927)	ClinicalTrials.gov
Xaluritamig (AMG 509)	STEAP1 × CD3	Half-life-extended BiTE antibody	Metastatic castration-resistant prostate cancer (mCRPC)	Intravenous infusion	Phase Ib (NCT05204999)	ClinicalTrials.gov
TNB-585/AMG 340	PSMA × CD3	Low-affinity CD3 bispecific	Metastatic castration-resistant prostate cancer	Intravenous infusion	Discontinued after Phase I (NCT04740034)	ClinicalTrials.gov
Ubamatamab (REGN4018)	MUC16 × CD3	Full-length bispecific antibody	Ovarian cancer	Intravenous infusion	Phase I (NCT03564340), multi-arm Phase II study in platinum-resistant ovarian cancer (NCT06787612)	ClinicalTrials.gov

Sources: FDA and EMA product information for approved agents; clinicalTrials.gov and publicly available clinical data for investigational agents.

This review aims to provide a comprehensive overview of TCEs in cancer immunotherapy, including their mechanisms of action, clinical applications, and associated challenges. It also discusses various types of TCEs, including bispecific T-cell engagers (BiTEs), trispecific T-cell engagers (TriTEs), and dual-affinity retargeting proteins (DARTs), their success in treating hematologic malignancies, their emerging potential in solid tumors, and their comparison with CAR-T-cell therapies. Key challenges such as tumor heterogeneity, toxicity, and pharmacokinetic limitations are also addressed. Various strategies to increase the therapeutic potential of TCEs and future trends, including the role of synthetic biology and AI in designing next-generation TCEs and their potential applications beyond cancer, have also been examined.

It is important to note that throughout this review, the term TCE is used as an umbrella term encompassing all T cell-redirecting bispecific and multispecific formats, including BiTEs (which are scFv-based fragment formats without an Fc domain), BsAbs (which include IgG-like formats with an Fc domain), and ImmTACs (immune-mobilizing monoclonal TCRs against cancer, which use an affinity-enhanced TCR rather than an antibody-based binding domain). While these formats share the common goal of redirecting T cells to tumor cells, they differ in their molecular architecture, pharmacokinetic properties, and clinical applications, as detailed in the sections below.

## 2 Mechanism of action of TCEs

**Figure 2:**
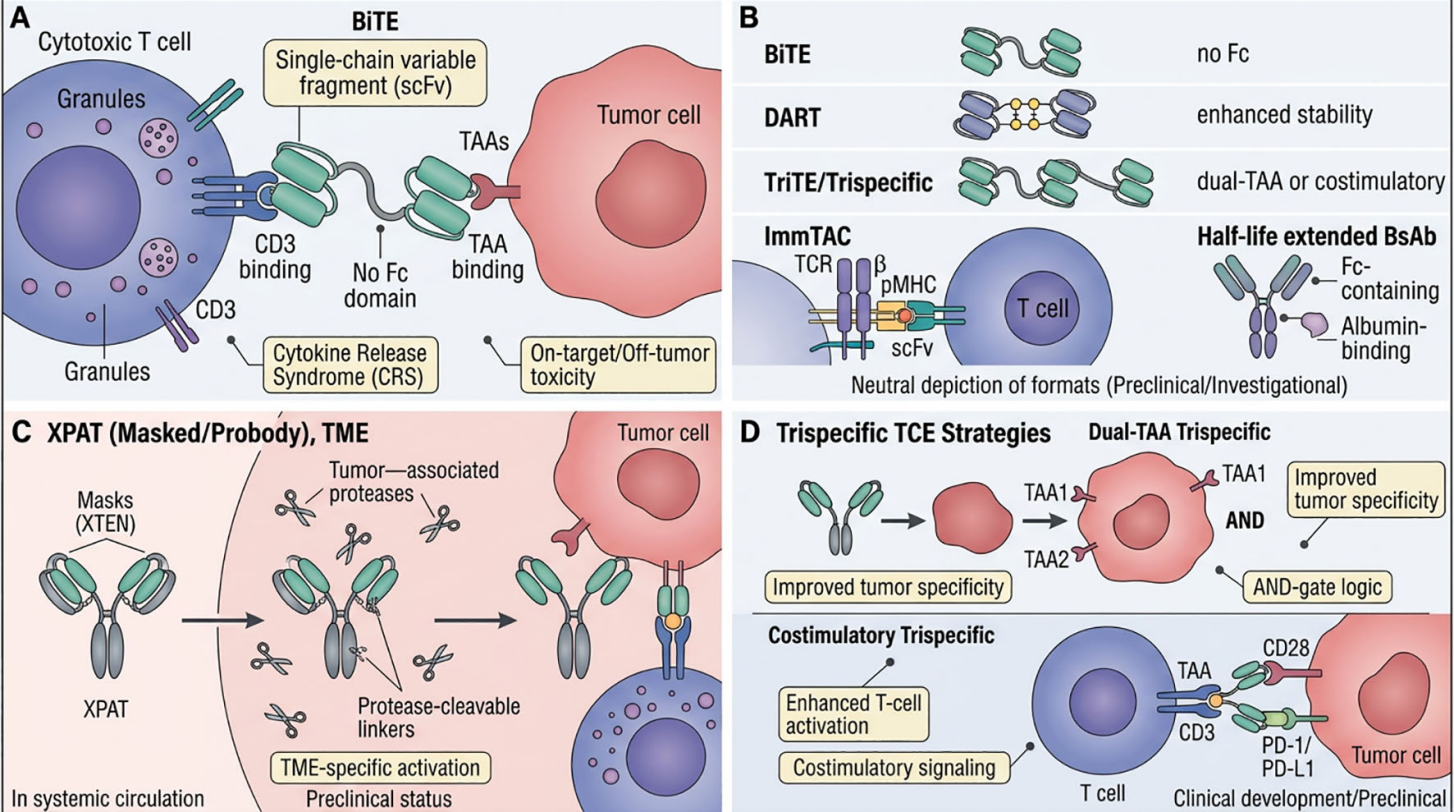
T-Cell engager therapy: mechanisms, formats, and next-generation strategies. **(A)** Bispecific T-Cell Engager (BiTE) mechanism: a cytotoxic T cell is linked to a tumor cell via single-chain variable fragments (scFvs) binding CD3 and a tumor-associated antigen (TAA), inducing cytotoxic granule release and tumor cell lysis. Key clinical considerations include cytokine release syndrome (CRS) and on-target/off-tumor toxicity. **(B)** Comparative overview of major TCE formats: BiTE (no Fc domain), DART (enhanced stability via disulfide bonds), TriTE/Trispecific (dual-TAA or costimulatory third arm), ImmTAC (affinity-enhanced TCR fused to anti-CD3 scFv targeting peptide-MHC complexes), and half-life extended BsAb (Fc-containing or albumin-binding formats). **(C)** XPAT (XPect Protease-Activated TCE) mechanism: XPAT constructs are masked by XTEN domains in systemic circulation and are selectively activated by tumor-associated proteases via protease-cleavable linkers within the tumor microenvironment, enabling TME-specific T cell activation while remaining inert in healthy tissues. **(D)** Trispecific TCE strategies: Dual-TAA Trispecific constructs engage two tumor-associated antigens simultaneously using AND-gate logic to improve tumor specificity and reduce immune escape; Costimulatory Trispecific constructs engage CD3 and a TAA while simultaneously providing costimulatory signaling via CD28 or blocking PD-1/PD-L1, enhancing T cell activation and overcoming immune suppression.

TCE activity depends on the engagement and activation of the TCR/CD3 complex. This complex is a multimeric structure composed of a variable αβ heterodimer (TCRα/TCRβ), which recognizes the peptide–major histocompatibility complex (MHC), and three invariant CD3 dimers, CD3γϵ, CD3δϵ, and CD3ζζ, arranged in a fixed stoichiometry. While TCRα/β recognizes MHC-presented polypeptides, the CD3 subunits trigger intracellular signaling cascades through their immunoreceptor tyrosine-based activation motif (ITAM), leading to T-cell activation, proliferation, and the release of cytokines, mainly cytotoxic granzymes and perforin (12). TCEs bypass native antigen recognition by directly engaging the CD3 complex and simultaneously redirecting T cells toward tumor-associated antigens in an MHC-independent manner. Most BsAbs enhance cytotoxicity by binding to the CD3ϵ subunit, which is shared by both CD3γϵ and CD3δϵ dimers and tumor antigens, thereby forming an immunological synapse between T cells and tumor cells that bypasses the need for MHC restriction. These BsAbs are classified into two formats: small fragment-based forms without an Fc domain (e.g., BiTEs) and IgG-like formats, which include engineered Fc domains to improve stability and extend serum half-life (13). However, since IgG-like TCEs retain an Fc region, specific modifications are required to prevent unintended immune activation. The most commonly employed modifications include the LALA mutation (L234A/L235A), which significantly reduces Fc gamma receptor (FcγR) binding, and the LALAPG mutation, which additionally abrogates C1q-mediated complement activation (14). Another frequently used approach is the N297A or N297G substitution, which eliminates Fc N-glycosylation and thereby reduces FcγR engagement. Together, these modifications effectively silence Fc-mediated immune effector functions while maintaining the structural integrity of the antibody (15). By bridging tumor cells and T cells, TCEs effectively trigger T-cell activation and promote targeted tumor cell destruction (16) (**Figure 2**).

### 2.1 Structure and function of TCEs

#### 2.1.1 Bispecific T-cell engagers

BiTEs are engineered antibodies that contain two distinct binding domains. One domain specifically targets tumor-associated antigens, such as B-cell maturation antigen (BCMA), CD19, or δ-like protein 3 (DLL3), whereas the other domain targets CD3 on T cells. These two domains consist of single-chain variable fragments (scFvs) derived from monoclonal antibodies connected by a flexible peptide linker. The first scFv domain can be modified to target a wide range of surface antigens, allowing for off-the-shelf therapies that can be used immediately for retreatment. The second scFv binding domain is universally specific for CD3. When the BiTE molecule binds to both a cytotoxic T-cell and a tumor cell, it stimulates T-cell proliferation, increasing the number of effector cells and increasing therapeutic efficacy. This interaction leads to the destruction of malignant cells. Since this process bypasses the need for co-stimulation or MHC presentation, BiTE can activate any T-cell regardless of patient-specific factors (17).

##### 2.1.1.1 Clinical advances and resistance in BiTE therapy

Various formats of BiTE molecules exist. The first approved BiTE, blinatumomab (18), is a BsAb composed of two scFvs linked together (19). In the pivotal TOWER trial, blinatumomab demonstrated significantly longer overall survival compared with standard chemotherapy in adults with relapsed or refractory B-cell precursor ALL, with a median OS of 7.7 versus 4.0 months, and a complete remission rate of 34% versus 16% with chemotherapy (20). Another common format is the IgG-like BiTE, such as teclistamab (21), which was recently approved for multiple myeloma treatment in the US and EU. Talquetamab, which is also IgG-like, demonstrated a 70% response rate in a phase 1 trial involving heavily pretreated multiple myeloma patients and is on an expedited development path in the US (22). BiTEs have proven effective in treating patients with hematologic malignancies. By binding both T cells and tumor cells through their unique structure, BiTEs increase tumor lysis and provide viable treatment options for patients with relapsed or refractory cancers, regardless of mutation or T-cell dysfunction. Ongoing advancements in molecular design, dosing regimens, and combination therapies have the potential to further improve the efficacy and safety of BiTEs. Innovative platforms are facilitating the development of new bispecific T-cell-recruiting antibodies with higher affinity, greater flexibility, and longer half-lives. The efficacy and toxicity of these emerging agents are being assessed in clinical trials. Further investigations into combination therapies with PD-1/PD-L1 blockers are expected to effectively prevent tumor escape. This work is anticipated to drive the evolution of antitumor strategies centered on BiTEs (23).

##### 2.1.1.2 Mechanisms of resistance and evolution to trispecifics

As with other cancer treatments, tumors can develop resistance to BsAbs/BiTEs, reducing their therapeutic effectiveness. These agents apply selective pressure on tumor clones expressing the target antigen, which can promote the outgrowth of subclones lacking that antigen, resulting in antigen escape and therapy resistance. To solve this problem, strategies involving multiple BsAbs or TsAbs targeting an additional tumor antigen have been investigated (24). Additionally, specific genetic abnormalities in acute myeloid leukemia (AML) and acute lymphoblastic leukemia (ALL) have been linked to weaker responses to BsAbs/BiTEs, although the exact mechanisms remain unclear. Tumor cells may also resist T-cell redirection by modifying intracellular signaling pathways, such as those involving interferon-gamma, in HER2-positive tumors. Additionally, as reviewed by Omer et al., factors outside the tumor, including the strong presence of regulatory T cells in the TME, have been shown to modulate the therapeutic response to BsAb/BiTE in multiple myeloma and B-cell acute lymphoblastic leukemia (B-ALL) (24). Resistance may also emerge due to prior cancer treatments that impair T-cell function. Long-term BiTE administration can overstimulate T cells, leading to exhaustion and tumor survival. Although blinatumomab has demonstrated clinical success in treating B-cell malignancies, challenges such as inconvenient administration methods, resistance to therapy, and limited effectiveness in solid tumors persist. To address these issues, significant efforts have focused on modifying the structure of traditional BiTEs and creating multifunctional T-cell engagers, some of which have already progressed into clinical development (6). These resistance mechanisms highlight the therapeutic limitations of conventional BiTEs and provide the primary rationale for the development of trispecific and multispecific engager formats, which are discussed in detail in Section 2.1.2.

#### 2.1.2 Trispecific engagers (TriTEs)

TsAbs are designed to target multiple antigens simultaneously, increasing immune response specificity and reducing the risk of immune escape (25). A study conducted by Antonio Tapia-Galisteo et al. (2022) explored the development and application of TsAbs in immunotherapy, with a focus on enhancing immune cell function against cancer by improving tumor penetration. Smaller TsAb formats incorporate scFvs and variable heavy-chain domains (VHHs). Key designs include checkpoint-inhibitory T-cell engagers (CiTE), triple bodies, trispecific killer engagers (TriKEs), and trispecific T-cell activating constructs (TriTACs), each employing distinct immune activation mechanisms (25).

Among these, TriTEs are notable classes with three binding arms, one that targets T cells via CD3 and another that recognizes a TAA. The third arm confers unique functional properties by enhancing immune synapse formation and improving immune cell activation (11). One such example is SAR-442257, which targets CD3, CD28, and CD38. CD3 recruits and activates T cells, whereas CD28 provides co-stimulation and helps prolong the immune response (26). A first-in-human phase I trial evaluated the safety, efficacy, pharmacokinetics (PK), and pharmacodynamics (PD) of SAR442257 in patients with relapsed and refractory multiple myeloma (RRMM) and relapsed/refractory non-Hodgkin lymphoma (rrNHL) (27).

TriTEs have also been engineered to engage two TAAs on solid tumors to improve T-cell activation and minimize immune escape. One such design targets epidermal growth factor receptor (EGFR), epithelial cell adhesion molecule (EpCAM), and membrane-bound surface proteins alongside CD3 to minimize immune escape and reduce off-target toxicity. In parallel, trifunctional NK cell engagers have been developed to bind the activating receptors NKp46 and CD16 on NK cells while also targeting a TAA. Another example includes a TsAb directed at CD38, CD3, and CD28, further boosting T-cell activation and tumor targeting. These Fab-based constructs include Fc regions but often require Fc modifications to prevent unintended immune activation (25).

In colorectal cancer, EGFR is a primary target, but resistance limits the effectiveness of monoclonal antibodies such as cetuximab. EpCAM, which is widely expressed in carcinomas, contributes to tumor cell adhesion, proliferation, migration, and invasion. Although Catumaxomab, an EpCAM × CD3 IgG bispecific antibody, was previously approved, it was later withdrawn in 2017 because of Fc-mediated T-cell activation, resulting in hepatotoxicity (25).

### 2.1.3 Dual affinity retargeting molecules

DARTs, similar to BiTEs, feature a disulfide bridge to increase the stability of the molecular complex (28). They are engineered by connecting the variable light (VL) and variable heavy (VH) domains of one antibody with those of another, forming heterodimeric polypeptide chains (29). These chains are arranged in a VH-linker-VL configuration and heterodimerize through disulfide bonds between cysteines at their C-termini. Typically, DARTs are produced by co-expressing two chains, such as VLA-linker-VHB-tail and VLB-linker-VHA-tail, in a host cell (30). DARTs are more stable because disulfide bonds link polypeptide chains and attach to an Fc domain, which extends their half-life to approximately 6.7 days (31). Flotetuzumab (MGD006) is an investigational CD123 × CD3 DART^®^ developed by MacroGenics that redirects CD3+ T cells to eliminate CD123+ cells (32). Preclinical studies have shown that DART molecules exhibit superior cytotoxicity compared with conventional BiTEs. These constructs can redirect both NK cells and T cells in models of hematologic malignancies, such as anti-CD16-CD32B and anti-CD19-CD3 DARTs (e.g., duvortuxizumab/MGD011), as well as in solid tumors, including gastrointestinal cancers expressing glycoprotein A33 (gpA33), targeted by MGD007 (33).

#### 2.1.4 Other novel formats in preclinical or clinical trials

##### 2.1.4.1 Trispecific killer cell engagers

TriKEs are trifunctional single-chain molecules designed to bind a TAA with one arm, CD16 with another, and incorporate interleukin-15 (IL-15) to enhance the activation of recruited NK cells. GTB-3550 is a fusion protein consisting of anti-CD16 and anti-CD33 scFvs flanking human IL-15. In an AML model, GTB-3550 demonstrated greater *in vivo* efficacy than the corresponding bispecific killer engager lacking IL-15, along with prolonged persistence of human NK cells (11).

TriKEs are designed to target specific TAAs and bypass immune checkpoints. They selectively activate NK cells, leading to a targeted immune response against cancer cells without inducing T-cell hyperactivation, as seen in ICIs. NK cells are less likely to cause cytokine storms and off-tumor effects; hence, TriKEs represent a promising option for cancer immunotherapy (34).

##### 2.1.4.2 Trispecific T-cell activating constructs

TriTACs are next-generation T-cell engagers with three functional domains: one targeting a tumor antigen, another binding to human serum albumin to extend the half-life, and a third binding to the CD3ϵ subunit. The tumor and albumin-binding domains are typically humanized single-domain antibodies derived from camelid heavy-chain-only antibodies (35). TriTACs are currently under evaluation in several phase I/II trials for solid tumors. One example is HPN424, which targets prostate-specific membrane antigen (PSMA) in prostate cancer (Trial ID: NCT03577028) (36). HPN424 is engineered as a compact, globular protein to facilitate deep penetration into solid tumors and prolong circulation time (37).

Another example, HPN328, is designed to effectively guide T cells to target and destroy DLL3-positive small cell lung cancer (SCLC). In immunocompetent mice engineered to express the human CD3ϵ epitope (hCD3ϵ), HPN328 triggered strong antitumor responses and established durable immune memory, as demonstrated by successful tumor rechallenge. Preclinical evaluations in cynomolgus monkeys revealed that HPN328 has favorable safety and pharmacokinetic profiles (38).

##### 2.1.4.3 Tandem antibodies

Tandem diabodies (TanAbs) are tetravalent antibody constructs formed by connecting two scFvs in a head-to-tail fashion. This configuration creates a molecule with dual specificity and bivalent binding for each target, thereby enhancing functional avidity (39). AFM11 is a humanized, tetravalent bispecific antibody in TandAb^®^ format developed for targeting CD19-positive non-Hodgkin lymphoma (NHL) and ALL. It binds simultaneously to CD19 on B cells and to CD3 on T cells, facilitating the formation of an immunological synapse that activates T cells and induces apoptosis in malignant B cells. *In vitro* data indicate that, compared with blinatumomab, AFM11 has an approximately 100-fold greater affinity for CD3, suggesting the potential for enhanced therapeutic efficacy (40).

Additional T-cell engager formats have been introduced, including bifunctional CiTEs and simultaneous multiple interaction T-cell engagers (SMiTEs). CiTEs are designed to overcome resistance mechanisms associated with traditional BiTE therapy, particularly those driven by the upregulation of immune checkpoints such as PD-1/PD-L1. They incorporate a low-affinity extracellular PD-1 domain to competitively inhibit PD-1/PD-L1 binding (41). In contrast, SMiTEs are composed of two BiTE molecules: one engages a TAA and CD3, while the other targets CD28 and either the same antigen or an alternate one, such as PD-L1. This design effectively transforms an immune checkpoint receptor into a source of costimulatory signaling (42).

## 3 Clinical applications of TCEs

### 3.1 Hematologic malignancies and early approvals

The therapeutic potential of TCEs has been demonstrated with the approval of blinatumomab for B-cell precursor acute lymphoblastic leukemia, which is now being increasingly recognized in oncology (43). Hematological malignancies are cancers of the myeloid and lymphatic systems that result from disruptions in the normal process of blood cell development (44). TCEs represent a significant breakthrough in the management of B-cell and plasma cell malignancies and are also being explored as promising approaches for solid tumors (45). This growing recognition is reflected in recent regulatory approvals, such as teclistamab for multiple myeloma and mosunetuzumab for follicular lymphoma, reinforcing their clinical value in hematologic malignancies.

#### 3.1.1 Teclistamab

Teclistamab is a humanized bispecific IgG4-PAA antibody that links CD3 on T cells to BCMA on malignant B cells, triggering T-cell-mediated cytotoxicity in BCMA-positive cells (46). It is the first bispecific T-cell engager targeting both BCMA and CD3 approved for treating RRMM in patients who have undergone at least three prior therapies, including an immunomodulator, a proteasome inhibitor, and an anti-CD38 agent (47). In the phase I/II MajesTEC-1 trial, teclistamab achieved an overall response rate (ORR) of 63% in triple-class refractory myeloma patients. The median progression-free survival (PFS) was 11.3 months, and 39.4% of patients achieved a complete response or better. The median duration of response was 18.4 months. CRS was observed in 72.1% of patients, but was predominantly grade 1 or 2 (48).

#### 3.1.2 CD20 × CD3 bispecific antibodies in lymphomas

Follicular lymphoma (FL) is an indolent subtype of non-Hodgkin lymphoma arising from germinal center B cells. It is among the most common subtypes of slow-growing B-cell NHLs, primarily affecting older adults, with an annual incidence of 3–5 cases per 100,000 individuals annually in Western countries (49, 50).

Mosunetuzumab (Lunsumio^®^), a CD20xCD3 bispecific antibody developed by Genentech (Roche), is administered to outpatients and has demonstrated favorable safety and efficacy profiles in patients with relapsed/refractory FL and diffuse large B-cell lymphoma (DLBCL), including patients previously treated with CAR-T-cell therapies. In a pivotal trial (Trial ID: NCT02500407), the ORR was 42.0%, and the complete response rate was 23.9% (51, 52).

Epcoritamab, another bispecific antibody that targets CD3 and CD20, is administered subcutaneously and is approved for treating adults with relapsed or refractory large B-cell lymphoma (LBCL), including DLBCL and FL, after two or more prior systemic therapies (53). The EPCORE NHL-1 phase I/II trial (Trial ID: NCT03625037) showed durable responses and a median overall survival (OS) of 18.5 months, demonstrating a manageable safety profile.

Glofitamab, another intravenously delivered CD3xCD20 bispecific antibody, is approved for third-line treatment of relapsed or refractory DLBCL or LBCL, with a reported median OS of 11.5 months (54). Economically, glofitamab offers per-patient cost savings over epcoritamab at each treatment cycle owing to its fixed 12-cycle regimen, less frequent dosing, and lower drug acquisition costs, despite higher administration and AE management expenses. Across all time horizons (1-, 5-, 10-year, and lifetime), it maintains a lower total cost and improved budget predictability (55). In support of these findings, a hypothetical 1,000,000-member health plan that introduces glofitamab into the third-line DLBCL market could save $728,697 in total costs and $0.0202 per member per month over three years (56).

### 3.2 Solid tumor applications

Hematologic cancers have traditionally been excluded from basket trials, which typically focus on solid tumors. Increasing evidence indicates that both cancer types can harbor common driver mutations and actionable surface markers. Alterations such as BRAFV600E, ALK, FGFR1, NTRK fusions, IDH1/2 mutations, and PD-L1 amplification are common across malignancies and may respond effectively to targeted or immune therapies (57).

Tebentafusp represents a distinct class of TCE known as an ImmTAC, comprising an affinity-enhanced T cell receptor fused to an anti-CD3 scFv. Unlike conventional antibody-based TCEs, tebentafusp targets the intracellular antigen gp100 presented on HLA-A*02:01 molecules on the surface of uveal melanoma cells (58), making it the first TCR-based TCE to receive regulatory approval for use in the US and Europe (59, 60). In the pivotal phase III IMCgp100–202 trial, tebentafusp demonstrated a significant overall survival benefit compared with investigator’s choice therapy, with a median OS of 21.7 months versus 16.0 months for standard of care. Notably, this survival benefit was observed regardless of objective radiological response, suggesting tebentafusp may modulate the tumor immune microenvironment beyond direct tumor cell killing (61). However, its applicability is restricted to HLA-A*02:01-positive patients, limiting its use to approximately 45% of persons in the US and Europe (61).

Tarlatamab is a half-life extended BiTE that simultaneously targets DLL3 on tumor cells and CD3 on T cells, leading to T cell-mediated tumor lysis in an MHC-independent manner. DLL3, an inhibitory Notch ligand, is aberrantly expressed on the surface of SCLC cells in up to 85 to 94% of patients while remaining minimally expressed in normal tissues, making it a compelling therapeutic target (62). Tarlatamab received FDA approval in May 2024 for extensive-stage SCLC following progression on prior therapy, representing the first TCE approved for this indication (63). In the pivotal phase II DeLLphi-301 trial, patients previously treated with SCLC received tarlatamab at either 10mg or 100mg every two weeks. The 10mg dose demonstrated a superior benefit-to-risk profile, achieving an objective response rate of 40%, with 59% of responders maintaining response for at least 6 months and a 9-month overall survival estimate of 68%. CRS was observed in 51% of patients in the 10 mg group but was predominantly grade 1 or 2 in severity and largely confined to the first treatment cycle (63).

HPN424 is a novel, first-class, trispecific T-cell engager derived via the TriTAC platform (64). It has three distinct binding domains: an anti-PSMA (prostate-specific membrane antigen) domain that targets prostate-specific membrane antigens expressed on tumor cells, an anti-albumin domain that binds to serum albumin to extend the circulatory half-life, and an anti-CD3 domain that engages and activates T cells to mediate cytotoxicity (37). It is designed as a small, globular protein to improve its ability to infiltrate solid tumors and improve pharmacokinetics (37). In the first-in-human phase 1 dose escalation study in heavily pretreated mCRPC patients, HPN424 demonstrated a manageable safety profile with pharmacodynamic evidence of T-cell activation and PSA reductions, with 8 patients remaining on study for more than 24 weeks (65).

Xaluritamig (AMG 509) is a STEAP1 x CD3 bispecific TCE featuring two STEAP1-binding domains designed to exploit avidity-driven selectivity for tumor cells with high STEAP1 expression, a cell-surface antigen expressed in 77-83% of prostate cancer metastases with limited expression in normal tissues. In the first-in-human phase I dose escalation study in 97 heavily pretreated mCRPC patients, xaluritamig achieved a PSA50 response rate of 49% and an ORR of 24% overall, rising to 59% PSA50 and 41% ORR at target doses >0.75mg. CRS occurred in 72% of patients but was predominantly low grade and confined to cycle 1, improving with premedication and step dosing (66). Despite such advances, solid tumors remain challenging for T-cell engager therapies because of their immunosuppressive microenvironment, heterogeneous antigen expression, and lack of truly tumor-specific surface markers (12).

Additional emerging TCE targets in solid tumors under clinical investigation include B7-H3 (67), HER2 (68), CLDN18.2 (69), and EpCAM-masked constructs, reflecting the expanding antigen landscape for TCE-based immunotherapy in solid cancers (70).

### 3.3 Comparative evaluation of TCEs and CAR-T-cell therapies

#### 3.3.1 Neurotoxicity and cytokine release syndrome

Neurotoxicity profiles differ notably between CAR-T cells and TCEs. Immune effector cell-associated neurotoxicity syndrome (ICANS) occurs in approximately 13–21% of patients receiving CAR-T-cell therapy and lasts 4–5 times longer than neurotoxic events associated with TCEs (71–73). Neurologic adverse events associated with TCEs, such as headache, are generally milder and occur less frequently than ICANS with CAR-T-cell therapy (74). CRS is the most frequent adverse effect of CAR-T-cell therapy, with incidence rates ranging from 42% to 100%, and severe manifestations occur in up to 46% of patients. It is triggered by robust immune activation and increased release of proinflammatory cytokines, including IL-1, IL-6, IFN-γ, and GM-CSF (75). In contrast, CRS associated with TCEs is typically limited to the first administration, especially during step-up dosing. Symptoms usually emerge within a median of 4–16 hours after infusion, with fever as the first and most prominent clinical symptom. However, since fever is not a specific symptom, alternative causes, particularly infections, should be carefully ruled out. CRS-related fever may be high grade and may persist for several days, but the overall frequency and severity of CRS and ICANS are significantly lower with TCE than with CAR-T-cell therapy (8).

#### 3.3.2 Clinical efficacy

Compared with BCMA TCE, BCMA-targeted CAR-T-cell therapy showed superior clinical efficacy in patients with RRMM who had previously been treated with BCMA-directed therapy. Specifically, the ORR was greater in the CAR-T-cell group (79% vs. 51%), as were the ≥very good partial response (VGPR) rates (64% vs. 47%). Moreover, the median progression-free survival (PFS) and OS were significantly longer in the CAR-T-cell cohort, i.e., 6 vs. 2 months for PFS and 30 vs. 12 months for OS. These findings highlight deeper and more durable responses with CAR-T-cell therapy (76). In contrast, AMG 420, a BCMA-targeted TCE, demonstrated lower rates of CRS in a phase I trial (NCT03836053), suggesting a potentially improved safety profile compared with BCMA-targeted CAR-T-cell therapies in RRMM (77).

#### 3.3.3 On-target, off-tumor toxicity

Despite their clinical promise, TCEs (especially BiTEs) target tumor cells via TAAs, but since many TAAs are also present on normal cells, they can cause on-target off-tumor toxicity. Some BiTEs have shown unacceptable toxicity in clinical trials (78). A notable example is acapatamab, a BiTE that targets both CD3 and PSMA. In a first-in-human trial involving patients with metastatic castration-resistant prostate cancer (mCRPC) (79), acapatamab induced modest antitumor activity in mCRPC, with a ≥ 50% PSA reduction in 31.6% of patients and a partial response in 10.6%. However, its clinical utility is limited by high rates of CRS (97.7%) and ocular toxicity (40.6%) due to its extended half-life (80).

Another well-known case is solitomab (MT110, AMG 110), a BiTE that targets EpCAM, an antigen that is overexpressed in various epithelial tumors but is also found in normal gastrointestinal tissue. Phase I trials of solitomab were terminated because of dose-limiting toxicity, including transaminitis and diarrhea, preventing the identification of a safe therapeutic window (81). This issue arises because many TAAs, although they are overexpressed in cancer cells, are also found at relatively low levels in normal tissues, which places healthy cells at risk. Strategies to mitigate on-target off-tumor toxicity through tumor-selective activation of TCEs, including protease-activated constructs such as XTENylated protease-activated T-cell engagers (XPATS) and conditionally bispecific-redirected activation (COBRAs), are discussed in detail in section 5.

#### 3.3.4 Cost and accessibility

For transplant-eligible patients with newly diagnosed multiple myeloma, the standard first-line treatment consists of a triplet regimen of lenalidomide, bortezomib, and dexamethasone, followed by autologous stem cell transplantation (82). While this regimen is clinically effective, the financial burden of these therapies is substantial. A typical regimen, such as lenalidomide plus dexamethasone, has an estimated drug cost of $330,000. The incorporation of bortezomib increases the cost to approximately $385,000, whereas the addition of daratumumab further increases the cost to approximately $627,000 (83). These figures do not include indirect costs, such as supportive care and monitoring.

In addition to frontline therapy, CAR-T-cell therapy represents an even greater economic challenge. As of 2023, in the United States, list prices for a single administration of CAR-T cells range from $424,000 to $543,828, with overall treatment costs potentially approaching $1 million when accounting for hospital stays and supportive care (84). Among immunotherapies, the FDA-approved BiTE blinatumomab, which is used for B-ALL, is priced at approximately $89,000 per course, representing a significantly lower-cost alternative to CAR-T-cell therapy. In contrast, CAR-T-cell therapy, particularly in multiple myeloma, results in a significantly greater financial burden (77).

#### 3.3.5 Mechanistic and clinical distinctions

Both CAR-T-cell therapy and TCEs activate T cells to kill malignant plasma cells. However, their mechanism and clinical logistics differ substantially. CAR-T cells require personalized ex vivo genetic modification, expansion, and reinfusion of autologous T cells. For example, in some cases, before infusion, patients must also undergo lymphodepleting chemotherapy involving a combination of fludarabine and cyclophosphamide to enhance CAR-T cell engraftment and expansion (85). This could cause manufacturing delays, logistical complexity, and higher upfront costs. In contrast, TCEs such as BsAbs do not require ex vivo modification; instead, they harness the patient’s own T cells by simultaneously binding to CD3 and a tumor-associated antigen. These off-the-shelf therapies can target various tumor antigens and can be administered without delays in personalized manufacturing (86).

#### 3.3.6 Efficacy in hematologic vs. solid tumors

**Figure 3:**
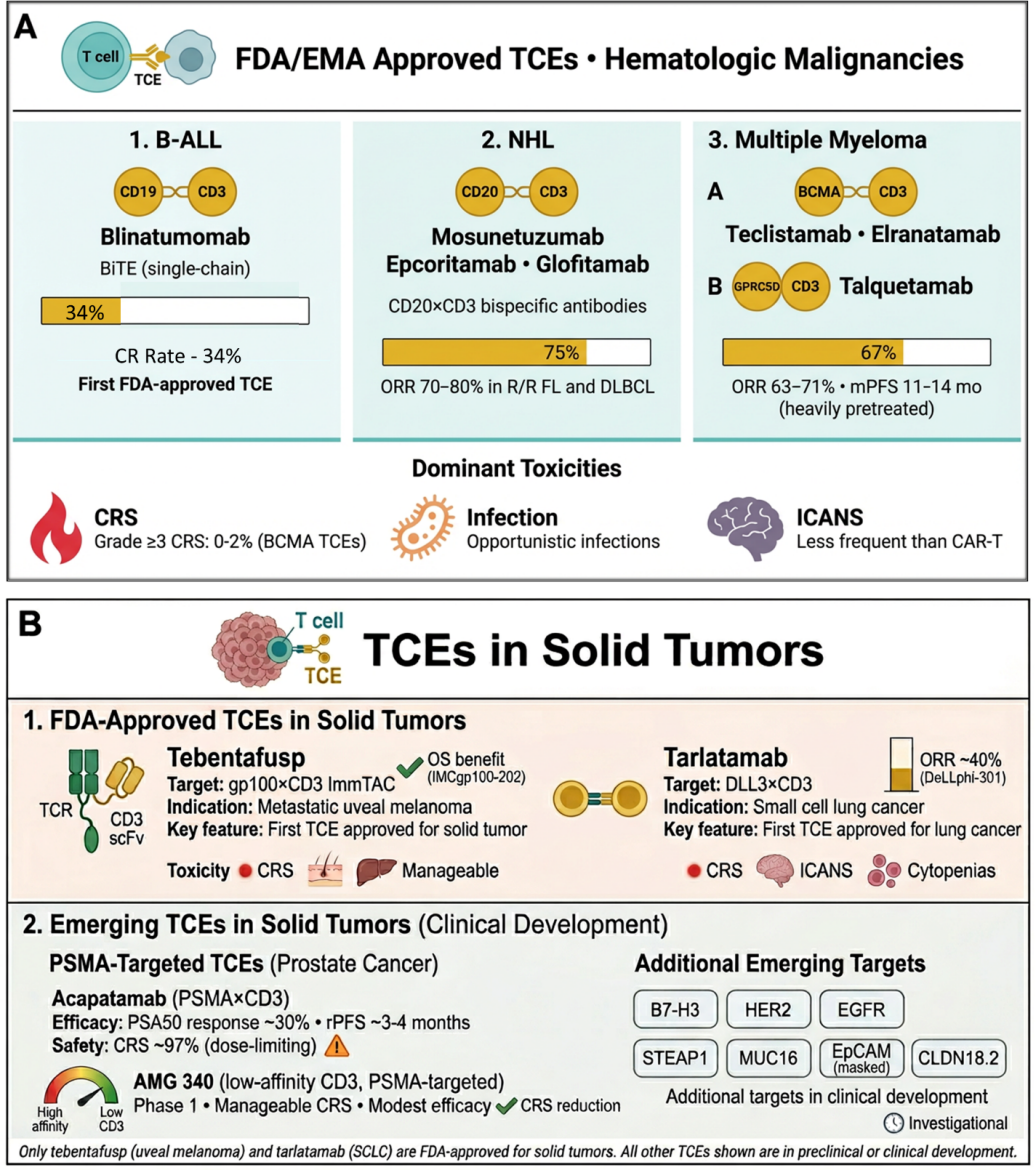
Clinical landscape of T-cell engagers (TCEs): approved therapies and emerging solid tumor applications. **(A)** FDA/EMA-approved TCEs for hematologic malignancies, organized by disease category: B-cell acute lymphoblastic leukemia (B-ALL), treated with blinatumomab (CD19×CD3 BiTE; CR rate ~34%; first FDA-approved TCE); non-Hodgkin lymphoma (NHL), treated with mosunetuzumab, epcoritamab, and glofitamab (CD20×CD3 bispecific antibodies; ORR 70–80% in relapsed/refractory FL and DLBCL); and multiple myeloma, treated with teclistamab and elranatamab (BCMA×CD3; ORR 63–71%, mPFS 11–14 months in heavily pretreated patients) and talquetamab (GPRC5D×CD3). Dominant toxicities across approved agents include CRS (grade ≥3: 0–2% for BCMA TCEs), opportunistic infections, and ICANS (less frequent than CAR-T cell therapy). **(B)** TCEs in solid tumors. FDA-approved agents include tebentafusp (gp100×CD3 ImmTAC; metastatic uveal melanoma; first TCE approved for solid tumors; OS benefit demonstrated in IMCgp100-202) and tarlatamab (DLL3×CD3; small cell lung cancer; first TCE approved for lung cancer; ORR ~40% in DeLLphi-301). Emerging investigational TCEs include PSMA-targeted agents acapatamab and AMG 340, as well as additional targets in clinical development including STEAP1, MUC16, HER2, EGFR, B7-H3, EpCAM (masked), and CLDN18.2.

CAR-T-cell therapy is more effective in hematologic malignancies than in solid tumors, in part because of their ability to circulate through the bloodstream and lymphatic system, allowing direct contact with blood-based tumor cells (87). In contrast, solid tumors are more difficult to treat because of limited T-cell infiltration and a restrictive tumor microenvironment. Although high response rates have been observed with TCEs targeting CD19, CD20, or BCMA in hematologic cancers, their success in solid tumors has been limited. To address this, a novel format called TriTAC (tri-specific T-cell-activating construct) has been developed, featuring high structural stability, a small molecular size, flexible linkers, a long serum half-life, and potent and specific T-cell-mediated cytotoxicity to improve safety and efficacy in solid tumors (88). The clinical application of T-cell engager is presented in **Figure 3**.

## 4 Challenges and limitations of TCEs

### 4.1 Toxicities and immunogenicity challenges

While TCEs have demonstrated strong antitumor activity, their clinical utility remains limited by various toxicities and immune-related complications. In addition to their cytotoxic potential, TCEs have shown encouraging antitumor effects, including prostate-specific antigen decreases and radiographic responses. However, their clinical application is hindered by immunogenicity, the formation of antidrug antibodies that may reduce drug exposure and therapeutic efficacy, and immune-related toxicities such as CRS (80).

### 4.2 Resistance mechanisms

Another important challenge is tumor resistance to T-cell engagers, as tumors may evade immune attack primarily by downregulating target antigen expression. Since TCEs activate T cells in an MHC-independent manner, resistance mechanisms involving disrupting HLA class I-mediated antigen presentation, which are relevant to conventional T cell therapies, are less applicable here. Instead, TCE-specific resistance pathways include antigen loss, T cell exhaustion, and immunosuppressive tumor microenvironment factors, which may arise from genetic mutations, transcriptional repression, or epigenetic modifications, ultimately leading to immune escape and treatment failure (45). These limitations highlight the need for next-generation TCEs to overcome these challenges (89).

### 4.3 Pharmacokinetic challenges and dosing limitations

TCEs such as blinatumomab and tebentafusp exhibit rapid systemic clearance from the body, which limits their dosing flexibility and therapeutic window (90). For example, blinatumomab, a small molecule of 55 kDa, lacks an Fc domain and does not bind the neonatal Fc receptor (FcRn), leading to fast elimination with a serum half-life of approximately 2 hours (89). Recent designs include half-life extension features such as Fc fragments or albumin-binding domains, enabling less frequent dosing (weekly or biweekly) while sustaining T-cell activation and therapeutic efficacy (91).

### 4.4 Tumor heterogeneity and antigen escape

Antigen loss, driven by tumor heterogeneity and the immune editing (81), is a key escape mechanism that compromises the therapeutic efficacy of T-cell-directed therapies, including TCEs (92). Recent evidence indicates that antigen escape via BCMA loss or mutation is a more common resistance mechanism to TCEs than previously thought. TNFRSF17 mutations were found in 42.8% of patients who relapsed after anti-BCMA TCE therapy, compared with 6% after CAR-T-cell therapy, suggesting stronger selective pressure from TCEs. Mutations such as p.Arg27Pro and p.Pro34del impaired TCE binding despite the presence of detectable surface BCMA, revealing that functional antigen escape was undetectable by standard assays. Not all therapies equally affect the retained activity of some bivalent or structurally distinct agents, underscoring the importance of TCE design and epitope targeting. These findings highlight the value of serial tumor profiling to identify emerging escape variants and inform treatment adjustments (93).

## 5 Strategies to overcome the limitations of T-cell engagers

### 5.1 Tumor-specific activation: XPAT proteins

To improve the therapeutic index of TCEs, tumor-specific activation strategies have been developed, most notably through the development of masked antibodies. These constructs remain inactive in circulation and are selectively activated by tumor-associated proteases within the tumor microenvironment. These constructs help reduce systemic toxicity and off-tumor effects, but typically require custom engineering for each antigen target (94). A novel class of such constructs is XPAT proteins, which incorporate protease-cleavable masking domains. By widening the therapeutic window, XPAT presents advantages over traditional monoclonal antibodies and antibody–drug conjugates (ADCs). Unlike conventional TCEs that remain active systemically, XPAT constructs are selectively activated by tumor-associated proteases within the TME, enabling targeted T cell engagement specifically at the tumor site while remaining inert in healthy tissues. Among these, EGFR-XPAT and HER2-XPAT are leading candidates and use the same masking and linker technology. EGFR-XPAT is currently under clinical evaluation (NCT05356741) (95). *In vitro* studies have demonstrated that protease-activated EGFR-XPAT and HER2-XPAT induce robust T-cell-mediated cytotoxicity (EC_50_: 1–2 pM), whereas their masked forms reduce off-target killing by 3,000–14,000-fold, underscoring the specificity and safety of this approach (96).

### 5.2 CD3 affinity tuning and PEGylation

Another emerging approach to improve TCEs involves modulating CD3 binding affinity, which plays a critical role in balancing tumor killing efficacy, cytokine release, and tissue distribution. Increasing CD3 affinity is associated with reduced T-cell-mediated killing, whereas lowering the affinity enhances T-cell activation and cytotoxicity, even at low concentrations (97). For example, JY108, developed via a patented PEGylated bispecific linker technology, targets CD19 and CD3ϵ. This design significantly reduces binding affinities to improve safety and address the limitations of current therapies (98). Compared with DS-8201a, a related construct, JY207 targets PD-L1 and CD47 via a 30k PEG-MMAE linker payload, resulting in improved tumor penetration, efficient internalization without efflux, no Fc-related toxicity, and superior tumor inhibition in HER2/PD-L1/CD47-positive models (99).

### 5.3 Multifunctional constructs

Novel platforms and combination therapies are emerging to increase the efficacy and personalization of TCEs (100). Among these innovations are CiTEs, which are built on the BiTE scaffold by adding an immunomodulatory protein. This addition helps overcome cancer cell-mediated immune suppression, thereby increasing therapeutic effectiveness. With continued advancements across diverse platforms, ICEs are showing increasing promise as scalable and personalized cancer treatments (101).

Relapsed/refractory AML and high-risk myelodysplastic syndrome (MDS) remain difficult to treat, especially in patients without actionable mutations or those who have exhausted standard treatment. While NK cell infusions following lymphodepleting chemotherapy have shown the potential to induce remission, they lack antigen specificity (102). To overcome this, GTB-3550 TriKE, a CD16/IL-15/CD33 tri-specific engager, was developed to combine NK cell-based therapy with targeted engagement for enhanced efficacy. Early studies demonstrated promising antitumor activity, prompting a phase I/II clinical trial. Clinical data have shown that NK cell activation occurs as early as day 3, with peak proliferation by day 8, and consistent NK cell expansion is observed at the end of the dosing cycle (103).

### 5.4 Biomarker-driven selection

Strategic biomarker-driven patient selection, coupled with rational combination regimens, is essential for maximizing therapeutic efficacy while minimizing resistance and treatment-related toxicity (104). Ongoing advances in tumor biology and immunology are paving the way for precision medicine in TCE therapy, where integrated biomarker panels combining genomic, antigenic, and immune profiling can guide target selection, predict response, and personalize treatment strategies (91). For example, the MajesTEC-1 trial (NCT04557098) incorporated BCMA expression as a selection biomarker to enroll patients eligible for teclistamab therapy among relapsed/refractory multiple myeloma patients (ClinicalTrials.gov).

## 6 Next-generation TCEs with enhanced specificity and potency

### 6.1 Tumor-selective activation mechanisms

The unique conditions of the tumor microenvironment allow for a conditional activation strategy that enhances the safety and efficacy of cancer-targeted therapies (105). In this approach, a therapeutic moiety (drug) is linked to a protease-labile linker, keeping the conjugate inactive until it is cleaved by the tumor-associated protease. This cleavage releases the active drug specifically within the tumor, minimizing systemic toxicity. This strategy has been successfully applied in the development of antibody-drug conjugates (ADCs), with two such compounds already approved for clinical use in cancer treatment (106).

One such platform, ProTriTAC, is a TCE prodrug (107) developed by Harpoon Therapeutics. It is built on a single polypeptide chain comprising three humanized antibody-derived domains: anti-albumin for half-life extension, anti-CD3 for T-cell engagement, and an anti-target antigen for tumor targeting (108). A masking moiety on the anti-albumin domain, linked by a protease-cleavable linker, blocks the CD3-binding site, keeping the prodrug inactive. In tumors, protease cleavage removes the mask, activating the drug to direct T-cell-mediated killing (109).

Conditionally activated bispecific TCEs offer the potential for an expanded therapeutic window by minimizing on-target effects in healthy tissues. They are designed to address the limitations of first-generation TCEs, which lack the ability to distinguish between tumor and healthy cells (110). COBRAs are a new class of TCEs specifically engineered to become active primarily within the TME (111). COBRAs bind cell surface antigens upon administration but activate T-cell engagement only within the TME. This design enables COBRAs to remain inactive in healthy tissues while being selectively activated in tumors, thereby improving safety and therapeutic precision (110).

Hypoxia is a common feature in approximately 50% of solid tumors, influencing tumor progression and resistance to therapy. However, current detection methods often overlook the complexity of the hypoxic tumor microenvironment, highlighting the need for further investigation (112, 113). Currently, hypoxia-conditioned CAR-T cells show promise in reducing on-target off-tumor toxicity in adoptive cell therapy (114). Similar strategies could shape the future development of TCEs to increase selectivity and safety in solid tumors.

### 6.2 Trispecific and multispecific TCEs

In the future, the development of TsAbs and their potential evolution into tetra-specific antibodies (TetraMabs) may represent the next frontier in TCE design (115). These multivalent and multispecific TCEs represent a next-generation cancer immunotherapy strategy aimed at improving efficacy by concurrently engaging multiple TAAs and immune pathways (100). In addition to improving tumor specificity and resistance mechanisms, such designs also exhibit improved pharmacokinetic and pharmacodynamic profiles, along with increased cytotoxicity against both tumor cells and HIV-infected cells (41).

### 6.3 Affinity optimization

The development of effective TCEs is limited by the intricate relationships among CD3 affinity, TCE format, TAA density, and target specificity. To overcome these hurdles, a novel TCE platform has been developed to fine-tune the binding affinity and structural format of CD3 for optimal alignment with TAA expression characteristics (116). An example of this approach is the human bispecific antibody REGN4018, which binds to both Mucin 16 (MUC16), a common diagnostic marker for various cancers (117), and CD3, thereby facilitating the recruitment and activation of CD3^+^ T cells against MUC16-expressing tumor cells (118). Another promising candidate, TNB-585, which features a low-affinity anti-CD3 domain, may offer effective treatment for prostate cancer while potentially reducing the incidence and severity of CRS compared with TCEs utilizing high-affinity anti-CD3 domains (119).

### 6.4 Role of synthetic biology and AI in optimizing TCE design

Synthetic biology and artificial intelligence (AI) have increasingly been integrated into the development of next-generation TCEs. AI has been utilized to predict immunotherapy responses by analyzing immune signatures, medical imaging, and histological data (120). Through large-scale data analysis, AI can uncover hidden patterns, anticipate patient outcomes, and aid in designing more personalized and effective treatment strategies (121).

A novel TCE model, the TROP2-targeting TCE, was developed with AI guidance and features both a CD3-targeting arm and a CD28-targeting arm. This dual-targeting design enables strong T-cell co-stimulation and significantly enhances T-cell-mediated tumor cell killing *in vitro* compared with versions with only a CD3-targeting arm (122). It operates at a level that preserves the bispecific antibody’s ability to inhibit tumor growth while significantly reducing Th1 cytokine secretion by T cells (123). Using logic gate principles, dual-input CAR-T cells and multispecific TCEs have been developed to improve cancer selectivity and efficacy in solid tumors by targeting two inputs instead of one. This may represent the best use of both technologies, where CAR-T cells act as cancer-homing vehicles that locally secrete TCEs, engaging endogenous T cells within the tumor microenvironment. These approaches, which are engineered with gated logic outputs and used alongside more selective and potent logic-gated TCEs, are likely to play a key role in future solid tumor therapies (124).

### 6.5 Expanding indications beyond current cancers

Recently, insights from the use of BsAb in cancer therapy have been successfully expanded to antiviral therapy. Virus-specific TCEs have been developed to redirect CD8+ T cells toward viral envelope proteins displayed on infected cell surfaces. This strategy has been explored for viruses such as HIV-1, cytomegalovirus, SARS-CoV-2, and hepatitis B virus (HBV) (125). Blinatumomab shows promise for expanded use beyond oncology. By linking CD3 on T cells to CD19 on B cells, blinatumomab facilitates targeted B-cell depletion. In addition to its effectiveness in treating hematologic cancers, its ability to lower immunoglobulin levels highlights its potential in treating B-cell-mediated autoimmune diseases, such as systemic sclerosis (126).

### 6.6 Combinatorial strategies: viral and cellular platforms

Oncolytic viruses have emerged as flexible platforms for developing innovative antitumor strategies, either as monotherapies or in combination with other immunotherapies (127). Oncolytic virotherapy is a treatment strategy that uses either naturally occurring or genetically modified viruses to specifically infect and eliminate cancer cells, leaving healthy tissues largely unharmed (128). One notable example is the oncolytic rhabdovirus vesicular stomatitis virus (VSVΔ51), which is engineered to express a truncated HER2 antigen (HER2T). This enables HER2-targeting with trastuzumab, converting HER2-negative tumors into HER2-mimics susceptible to trastuzumab emtansine, with demonstrated efficacy in *in vitro*, ex vivo, and *in vivo* models (129). Moreover, viral delivery of TCEs within the tumor microenvironment may bypass the short half-life of serum and off-target effects linked to systemic and localized production of TCEs (130). BiTE therapy for solid tumors is limited by poor penetration, a short half-life, and off-tumor effects, but combining it with OV therapy may overcome these challenges for more precise immunotherapy (78). In parallel, AgenT-797 is an innovative, allogeneic off-the-shelf iNKT cell therapy designed to elicit robust antitumor immunity across a range of cancer types (131). Combining next-generation ICIs or bispecific engagers with AgenT-797 enhances efficacy. AgenT-797 amplifies the activity of checkpoint modulators by activating T cells, decreasing T-regs, and stimulating dendritic cells. Furthermore, it can also be effectively engaged by clinical-stage engagers, resulting in more favorable activation and safety profiles than conventional T cells (132).

### 6.7 TCE-secreting T cells and CAR-T cells platforms

An emerging strategy to improve TCE delivery and therapeutic activity involves engineering T cells to constitutively secrete BiTE molecules, thereby enabling localized and sustained TCE production within the tumor microenvironment (133). These so-called engager T cells (ENG-T cells) combine the tumor-homing properties of adoptive T cell therapy with the bystander T cell recruitment capacity of TCEs, overcoming the pharmacokinetic limitations of systemically administered TCEs, such as short half-life and poor tumor penetration. In a foundational study, Iwahori et al. demonstrated that ENG-T cells secreting an EphA2xCD3 bispecific molecule efficiently redirected bystander T cells to EphA2-positive tumor cells, achieving potent antitumor activity in glioma and lung cancer xenograft models associated with a significant survival benefit compared with untreated controls (134). Similarly, ENG-T cells targeting CD123 demonstrated enhanced anti-AML efficacy in preclinical models. To incorporate an important safety mechanism, CD123-ENG T cells were further modified to express the CD20 suicide gene, enabling rituximab-mediated elimination of ENG-T cells without compromising anti-leukemic activity (135).

Building on this concept, CAR-T cells have been engineered to simultaneously express chimeric antigen receptors and secrete TCE molecules, creating a dual-function platform that combines antigen-specific CAR-mediated killing with TCE-driven recruitment of bystander endogenous T cells (136). This approach leverages CAR-T cells as tumor-homing vehicles that locally produce TCEs within the immunosuppressive tumor microenvironment, where systemic TCE delivery is often insufficient. Notably, engineered T cells secreting anti-BCMA TCEs have demonstrated potent control of multiple myeloma and promotion of immune memory in preclinical models (137). These combinatorial platforms represent a promising frontier in next-generation TCE design, with potential to address key limitations of both CAR-T cell therapy and conventional TCEs, including antigen escape, T cell exhaustion, and poor solid tumor penetration (124).

### 6.8 Emerging receptor-based formats: ImmTACs and TCR mimic antibodies

Beyond conventional antibody-based TCEs, two emerging receptor-based formats are advancing the field of T cell redirection. ImmTACs, exemplified by tebentafusp (discussed in detail in section 3.2), utilize affinity-enhanced TCRs fused to anti-CD3 scFvs to target intracellular tumor-associated peptide-MHC molecules, enabling recognition of antigens inaccessible to conventional antibody-based TCEs (58). Other ImmTAC molecules in clinical development include brenetafusp (IMC-F106C), which targets PRAME presented on HLA-A*02:01 and has demonstrated early antitumor activity in patients with cutaneous melanoma, with a Phase III trial now underway (138). TCR mimic (TCRm) antibodies represent a related approach, using antibody variable domains engineered to recognize peptide-MHC complexes with high specificity, thereby mimicking TCR antigen recognition while retaining the pharmacokinetic advantages of conventional antibodies (139). The first TCRm T cell engaging bispecific antibody to enter clinical trials, RO7283420, targets the WT1 peptide presented on HLA-A2 in relapsed/refractory AML and has demonstrated pharmacodynamic evidence of T cell activation in phase I evaluation (140). Together, these receptor-based formats significantly expand the targetable antigen space for T cell-redirecting immunotherapies beyond cell-surface proteins, representing an important frontier in next-generation TCE development (58, 139, 140).

## 7 Conclusion

TCEs have emerged as a transformative class of immunotherapies by redirecting cytotoxic T cells to tumor-associated antigens in an MHC-independent manner, offering promising clinical utility across hematologic and solid malignancies. This review highlights the evolution of TCEs from early BiTE formats to next-generation trispecific constructs, DARTs, TandAbs, and conditionally active designs such as XPATs and COBRAs. While agents such as blinatumomab and teclistamab have set benchmarks in hematologic cancers, barriers such as tumor antigen heterogeneity, immunosuppressive microenvironments, pharmacokinetic limitations, and on-target off-tumor toxicities continue to challenge TCE efficacy in solid tumors. Comparative analyses with CAR-T-cell therapy underscore the advantages of TCEs in terms of manufacturability, administration, and cost while also revealing trade-offs in terms of durability and toxicity. Future innovation lies in affinity optimization, multifunctional formats, and integration with synthetic biology, AI, and oncolytic virotherapy platforms. Expanding their utility beyond oncology into autoimmune and infectious diseases further highlights their versatility. Continued advancements in engineering, biomarker-driven targeting, and translational research are critical for unlocking the full therapeutic potential of TCEs in precision immunotherapy.
